# Aggressive behavior and recurrent spinal hydatid cyst

**DOI:** 10.11604/pamj.2021.40.202.32389

**Published:** 2021-12-06

**Authors:** Amine Trifa, Kais Maamri

**Affiliations:** 1 Fattouma Bourguiba University Hospital, Department of Neurosurgery, Monastir, Tunisia

**Keywords:** Hydatid cyst, spinal infection, spinal surgery

## Image in medicine

Hydatid cyst disease is caused by the parasite *Echinococcus granulosus*. Hydatidosis affecting the spine constitutes only 1% of cases. In 90% of the spinal cases, the disease is confined to the bone and the epidural space. Here we report a 25-year-old female patient, followed since the age of 3 for hepatic, renal and peritoneal hydatidosis, undergoing medical treatment (Albendazole). Operated 15 years ago (dorsal laminectomy) for spinal cord compression in consequence to the extension of the disease to the spinal cord at D12-L1 level. The course was marked by the progressive worsening of gait disorders with spinal deformity. The current examination shows a steppage gait and paraparesis, without sensitive disorders. The osteotendinous reflexes are abolished in the 2 lower limbs. We also noted the presence of a significant thoracolumbar kyphoscoliosis. A spinal MRI demonstrated a recurrence of hydatidosis centered on the vertebral body of D11 which is practically destroyed with right and left para-vertebral extensions at D10, D11, D12 and epiduritis in D11 (A, B, C, D). Spinal CT confirms the almost complete lysis of the D11 vertebral body (E). Albendazole (400 mg p.o.) is administered twice daily. A spondylectomy D11, curettage of the lesion and aspiration of necrotic tissue with spinal stabilization were proposed. Her situation remained unchanged after 4 months of follow-up. Although the hydatid cyst is characterized as benign pathology, in consequence of its local growing pattern, it can be classified in the malign group because of its high potential for dissemination, which can result in high incidence of recurrences.

**Figure 1:**
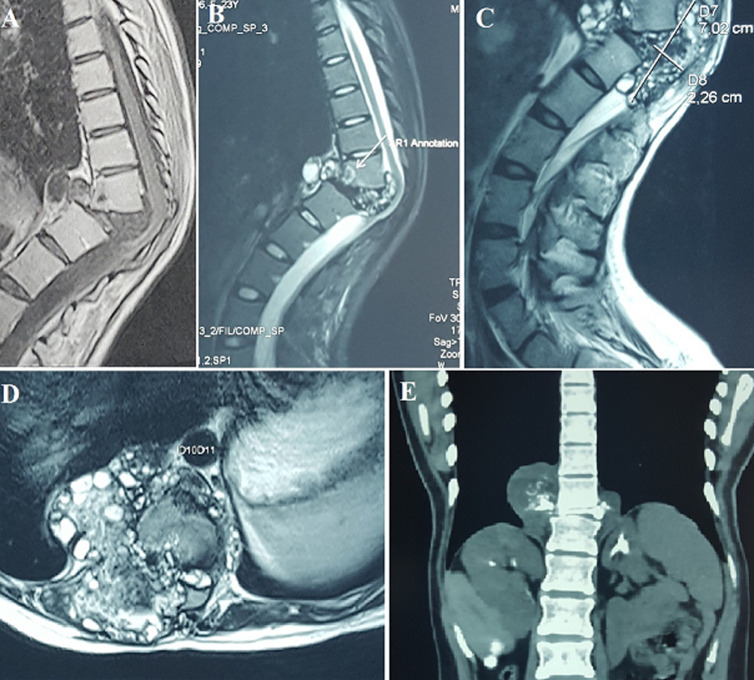
A) spinal MRI sagittal T1WI; B) sagittal T2WI; C) axial T2WI; D) frontal plane of CT spine; E) showing preoperative imaging

